# Modeling drug mechanism of action with large scale gene-expression profiles using GPAR, an artificial intelligence platform

**DOI:** 10.1186/s12859-020-03915-6

**Published:** 2021-01-07

**Authors:** Shengqiao Gao, Lu Han, Dan Luo, Gang Liu, Zhiyong Xiao, Guangcun Shan, Yongxiang Zhang, Wenxia Zhou

**Affiliations:** 1 Beijing Institute of Pharmacology and Toxicology, State Key Laboratory of Toxicology and Medical Countermeasures, Beijing, 100850 China; 2 School of Instrumentation Science and Opto-Electronics Engineering and Beijing Advanced Innovation Center for Big Data-Based Precision Medicine, Beihang University, Beijing, 100083 China

**Keywords:** MOA, Deep learning, Gene expression profiles, L1000

## Abstract

**Background:**

Querying drug-induced gene expression profiles with machine learning method is an effective way for revealing drug mechanism of actions (MOAs), which is strongly supported by the growth of large scale and high-throughput gene expression databases. However, due to the lack of code-free and user friendly applications, it is not easy for biologists and pharmacologists to model MOAs with state-of-art deep learning approach.

**Results:**

In this work, a newly developed online collaborative tool, Genetic profile-activity relationship (GPAR) was built to help modeling and predicting MOAs easily via deep learning. The users can use GPAR to customize their training sets to train self-defined MOA prediction models, to evaluate the model performances and to make further predictions automatically. Cross-validation tests show GPAR outperforms Gene set enrichment analysis in predicting MOAs.

**Conclusion:**

GPAR can serve as a better approach in MOAs prediction, which may facilitate researchers to generate more reliable MOA hypothesis.

## Background

Different drugs sharing similar gene expression signatures (perturbagens induced gene expression changes) may possess similar mechanism of actions (MOAs) [1]. Evaluating the drug-drug similarities at gene expression level can be used for drug repurposing [2]. Many methods or tools have been developed to compute drug-expression-signature similarities, but most of them evaluate the similarities by comparing differentially expressed genes (DEGs). For example, Gene set enrichment analysis (GSEA) [3] tools compare the most up/down regulated DEGs of one perturbation against the other perturbation signature [4–7]. The other widely used metrics to evaluate the perturbation correlations include cosine similarity, Jaccard score and *p* value of Fisher exact test between DEGs [8, 9]. However, drugs with shared MOAs may not present high similarity scores, because, firstly, drug-induced differential expression of the molecular target may be masked by the much larger differential expression of off-target genes [10], or even not related to nominal target gene perturbations [11], so limited up/down regulated DEGs may not cover focused MOAs; and secondly, the pairwise similarity evaluation may also be ineffective due to strong interferences such as batch effects [12] or common responses [13]. Compared to other methods available, deep learning, as a non-linear method excelling in fitting high-dimensional data, is independent of “feature” (MOA related genes) extracting, because the high layer structure of deep learning could suppress irrelevant variations [14]. Modeling MOAs using deep learning approach can not only reduce the influence of batch effects and noise with a large number of samples [15], but also facilitate to find the features closely related to drug MOAs.

As a matter of fact, a wide variety of machine learning methods have been developed to help to understand the mechanisms underlying gene expression [1, 16], and thus far some of previous published work has demonstrated that deep learning is an effective approach to connect signatures to prior knowledge such as side effects, indication, targets or drug sensitivity [1, 16–19], and has Noted that the high hidden layer feature of deep learning could effectively reduce the batch effect [15]. Considering the usage of the existing tools limited by the high cost in computational resource and the difficulty in model training and accuracy evaluation, here we report a new efficient method for querying gene expression data by taking advantage of deep neural networks to learn an embedding and do more precision classification. Moreover, in order to make the training, evaluation and prediction process easier for biologists and pharmacologists, we introduce the online tool called genetic profile-activity relationship (GPAR) implementing deep learning to easily model and predict MOAs. Users could train MOA models with self-defined training sets by simply providing the list of positive drugs or upload their own data. The GPAR here can also provide the accuracy evaluation via cross validation process to facilitate users evaluating the model performance and using the prediction results properly. Furthermore, by evaluating 103 MOAs, GPAR is demonstrated in real case scenarios to outperform the traditional approach.

## Methods

### Preparation of training set

We collect transcriptome data (GSE92742) from the LINCS dataset project achieved with L1000 platform [20], which is a high-throughput gene expression assay that directly measures the mRNA transcript abundance of 978 "Landmark genes" from human cells and infers the expression of 11,350 additional genes. It represents cellular responses to perturbation, such as drugs and RNAi, and was used to find relationships between diseases, genes, and therapeutics. The differential expression of directly measured 978 Landmark genes were computed by z-scoring procedure across all samples on the 384-Well Plate, where expression profiles were measured capturing most of the variance of whole-genome profiles at a much lower cost, and those expression data expressed by z-scores were used as input features, we also has tested how the size of features impacted the performance. we set the number of features N ranged from [10, 800]. In [10, 100], take 10 as the interval, and in [100,800], take 100 as the interval, and randomly extract N genes for 10 times to train and evaluate models, as shown in Additional file 1: Fig. S1, the area under the receiver operating characteristic curves (AUROC) increased with the size of features, and with the increasing size of training features, as shown in Additional file 1: Fig. S2.A–B, the similarity between shared MOAs has increased, as well as the distance between predefined positive and negative samples.

Then we reduced the sample size by selecting only one signature in each cell type for each molecule, Pearson correlation coefficient between each pair of signatures (within the same cell lines) was computed, and the one with the highest average correlation to the rest perturbation was regarded as the most representative signature. The binary classifier training set included 2 class samples: “positive set” and “negative set”. The “positive set” label was referenced by drug MOA annotation from both MCE library (https://www.medchemexpress.com/) and Drug Repurposing Hub [21], and then we removed some “positive” molecules whose signatures varied too much to the other “positive” set by repeating cross-validation process. We also selected 6220 compounds (with low transcript activity score [20] and without MOA annotation) as the invariant “negative set”, assuming they have no drug properties that can be reflected at transcriptome level.

### Training prediction models

Deep Neural network (DNN), one of the widely used deep learning architectures, was used to train MOA prediction models. The DNN was realized with the open source platform Tensorflow [22]. In order to choose appropriate hyperparameters, the hidden layers were tested from 2 to 5 and hidden nodes were tested from 10 to 2048 to find out the suitable ranges in which most prediction models have high AUROC and average precision score of precision-recall curve (AP score), and were robust to hyper parameters change (e.g. Additional file 1: Fig. S4). Finally, we chose 3 hidden layers containing 978, 512 and 256 nodes respectively, 2000 iterations, and used L1 regularization, RELU activator and *dropout* = 0.1. Here we note that deep learning has also been applied to the LINCS dataset to improve the accuracy of whole-genome expression from L1000 profiles and compute signatures [23], predict pharmacological properties of drugs [24], and map L1000 profiles to binary barcodes that improve prediction of compound structure and target information [15].

### Model evaluation and default model selection

*K*-fold cross-validation was used to evaluate the performance of prediction model. The value of *K* depends on the number of “positive” drugs (not signatures), that is, a “positive” drug should not be used as both training set and test set at the same time. Because when evaluating the performance of prediction model, we also want to know the consistence among drugs with the same MOA label via cross validation to get rid of drugs with low quality signatures or to further identify whether such MOA can reflect at transcriptome level or not.

The value of fold *K* equals to the “positive” drug number *N* if *N* ∈ [2, 4], *K* = 5 if *N* ∈ [5, 9], *K* = 10 if *N* ≥ 10, if *N* = 1, *K* equals to the number of signatures. During the cross validation, using the stratified sampling method to randomly divide the positive and negative drug set into *K* parts. (*K* -1)/ *K* samples were used as training sets to train a prediction model, evaluating its sensitivity and specificity by testing on the remaining 1/ *K* samples. This process would be executed *K* times, and *K* times mean *AUROC* were taken as the evaluating indicator, models with mean *AUROC* ≥ 0.6 were regarded as well trained models.

### Score all L1000 signatures via prediction model

The prediction classifier is not aiming for “classification” but for ranking the probability of each signature. Usually only the highest ranked (such as top10 or top50) prediction molecules may be worth further bioassay validation.

In order to predict new molecules that may share the same MOA, the prediction model would be used to score all the L1000 signatures (except the training set). The returned result was the probability that each signature was judged to be “positive”. As most molecules have more than one signature, the following enrichment statistic translated the probability rank orders of signatures into the enrichment score (*ES*) of each corresponding molecule. Supposing that the total number of all predicted signatures was n and the rank of compound *X* corresponded signature were ranked *R*(i), where i = 1, 2, 3, …, *k.* Then, compute the following values:

1 $$a = \mathop {\max }\limits_{i = 1,2,....,k} \left[ {\frac{i}{k} - \frac{R(i)}{n}} \right]$$

2 $$b = \mathop {\max }\limits_{i = 1,2,....,k} \left[ {\frac{R(i)}{k} - \frac{i - 1}{k}} \right]$$

The enrichment score of compound *X* was set to *a* if *a* > *b* or to − *b* if *b* > *a*. The high enrichment scores indicated all signatures of predicted compound *X* were enriched in top ranks. To evaluate the significance of enrichment score, we randomly drew instances for 1,000 repetitions to compute the corresponding enrichment score (*ES*_*i*_), where *i* = 1, 2, 3, …, N. The permutation *p* value was set to the frequency of *ES*_*i*_ < *ES*. Finally, we filter the results by following criteria: (1) the permutation *p* value of the molecule was less than 0.05; (2) the replicate samples of the molecule were more than 4. Then we rank results according to *ES*.

### Gene set enrichment analysis (GSEA)

Gene set enrichment analysis (GSEA) algorithm, a non-parametric, Kolmogorov–Smirnov statistic based similarity measure approach [3], was implemented via the *R* package GeneExpressionSignature [7] based on Iorio’s et al. method [25], which firstly merged multiple signatures of one molecule into one “optimal signature” by Borda Merging Function, and then 20 most up/down genes inside 978 directly measured “landmark genes” were selected to compute the enrichment score between each pair of signatures (positive and negative samples). After computing the GSEA similarity matrix, we rank the matches between pairwise signatures by enrichment scores, and the matches between the positive signatures and the positive signatures were set to true positive, while the matches between positive signatures and negative signatures were set to false positive, thereby drawing the receiver operating characteristic curve (ROC) and precision-recall curve (PRC).

## Results

### GPAR supports MOA discovery in two ways

The well-trained prediction models can be utilized in two ways: First, a certain prediction model can be used to search molecules that may share certain MOA by scoring all L1000 signatures, output is the rank of molecules (“Drug prediction” function). Second, multiple well-trained binary classifiers can be used to predict potential MOAs of input signatures, output is the rank of MOAs (“MOA prediction” function).

**Fig. 1:**
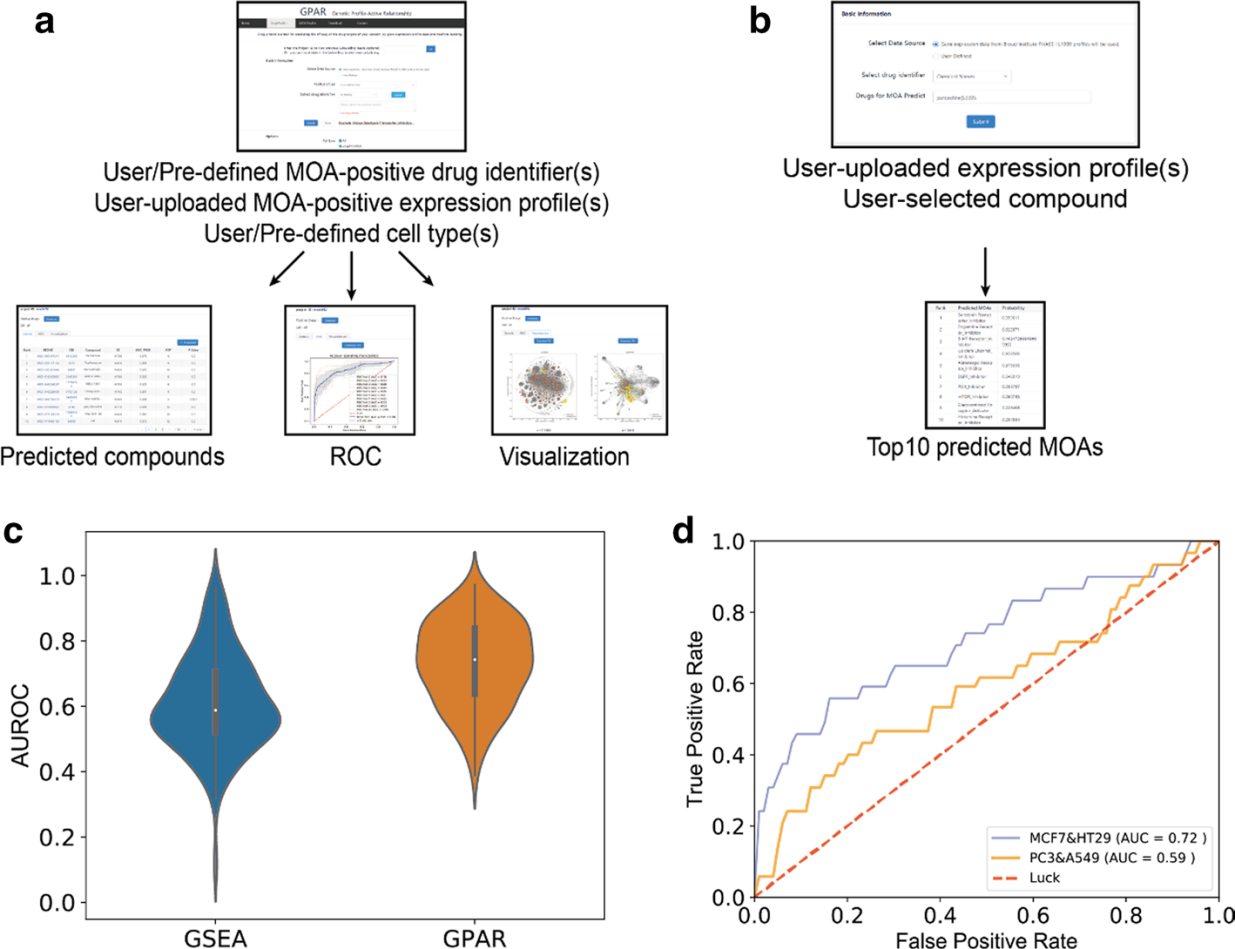
**a** Drugs prediction: user/pre-defined one or multiple drugs would be taken as positive samples in model training. And the predicted compound rank lists, *AUROC* of prediction model and visualizations of both training and predicted data would be returned. **b** MOA prediction: 83 MOA prediction models with *AUROC* ≥ 0.6 were used to predict the potential MOA of user uploaded or selected expression profiles. And the top 10 predicted MOAs would be presented. **c** The *AUROC* of GSEA and GPAR by calculating 103 MOAs. *AUROC* of GPAR is significantly higher than that of GSEA (Wilcoxon matched-pairs signed rank test, *p* < 0.0001). **d** Comparison of trained model performance of estrogen receptor agonists in PC3 and A549 (orange) cell lines and in MCF7 and HT29 (blue) cell lines

Drug prediction: The purpose of this function is to find molecules which may share the same MOA with user-defined “positive” molecules. As shown in Fig. 1a, one or multiple “positive” molecules (or user owned expression data) and the corresponding cell types are input to train prediction models. After the training, predicting and enrichment statistic processes, the output is the rank list of predicted molecules (training set were excluded), which could be downloaded in CSV format. Top predicted molecules will be listed, and the related information will be linked to the corresponding iLINCS (http://www.ilincs.org/ilincs/) and Pubchem entry (https://pubchem.ncbi.nlm.nih.gov/). We also provide *AUROC* of user trained prediction model, together with the data visualization of training set and top 30 predicted molecules in two visualization ways: L1000FWD and t-distributed Stochastic Neighbor Embedding (t-SNE) [26] implemented in scikit-learn library with default parameters [27].

MOA prediction: As shown in Fig. 1b, 83 MOA prediction models with high *AUROC* were used for quick MOA prediction. Users can select a molecule from L1000 platform or upload the CSV or TXT format file with expression data computed into z-scores, and the top10 predicted MOAs will be returned by computing the signatures’ average probabilities for each MOAs.

### GPAR serves as a better similarity measure

For calculating drug-drug similarities, though GSEA is a widely used method, we demonstrate the GPAR outperform GSEA by calculating *AUROC* of recovering 103 MOAs, as shown in Fig. 1c, Additional file 1: Table S1 and Fig. S3. The average *AUROC o*f GPAR (average *AUROC* = 0.73) was significantly (Wilcoxon matched-pairs signed rank test, *p* < 0.0001) higher than that of GSEA method (average *AUROC* = 0.61), showing the reliability of GPAR tool. Additionally, we also compared our work to Aliper’s work [28], noting that GPAR also has a better performance (Additional file 1: Fig. S5).

In order to benchmark DNN models with machine learning approach, we then compared DNN to three machine learning approaches including K-Nearest Neighbor (KNN), Random Forest (RF) and Naïve Bayes (NB) implemented in scikit-learn library with default parameters [27], as shown in Fig. Additional file 1: S6.A–B, the DNN also has achieved the highest performance.

### GPAR can be used to evaluate cell influence in model performance

One of the important factors that influence training process is the cell sources of expression data because of the specific distribution of drug targets [29]. Using different cell sourced expression data may got totally different model performances in discovering drug MOAs. For example, when training estrogen receptor (ER) agonist models, the prediction model trained with signatures from MCF7 and HT29 cells (ER expressing) achieved a higher *AUROC* compared to that trained with signatures from PC3 and A549 cells (no ER expressing) (Fig. 1d). Users can define the option to select different cells according to their needs with GPAR.

### Case study

Signatures of different MOAs have several differences: (1) some MOAs, especially most of the anti-cancer or cytotoxic property, have reproducible signatures and strong signature strength that can induce a large number of differentially expressed genes, and often companied with obvious phenotypic change such as apoptosis, whereas some MOAs have relatively mild properties and low transcript signals, such as serotonin transporter inhibitors. (2) Not all drugs with shared MOA may be highly consistent at transcriptome level, because some MOAs cannot be reflected at human cell transcriptome level [20] (e.g. anti-virus/bacteria), Gonçalves et al have systemically presented many diffculties in MOA researches, noting that not all drug are significantly correlated to nominal target gene perturbations [11], and it is therefore necessary to identify whether and the concerned MOAs are directly related to gene expression signatures or not. (3) For most of MOAs, their signatures change with cell types, time points and dosages, but there are still small parts of MOAs (e.g. Na+/K+_ATPase inhibitors) or toxic signatures [19] that were robust to those attributes. Therefore, difficulties in training different MOA prediction models are not equal. Generally, the MOAs with strong transcription signal, exhibit highly consistent signatures and the robustness to experimental attributes could be easily predicted by either GSEA (or any other widely used methods) or GPAR method. But our results showed that, for one, GPAR can be equal to or even outperform GSEA when predicting those easily trained MOAs. Besides, GPAR can still have good performance when predicting some MOAs whose signatures are inconsistent among differeent cell types, time points and pertubagen dosages.

**Fig. 2:**
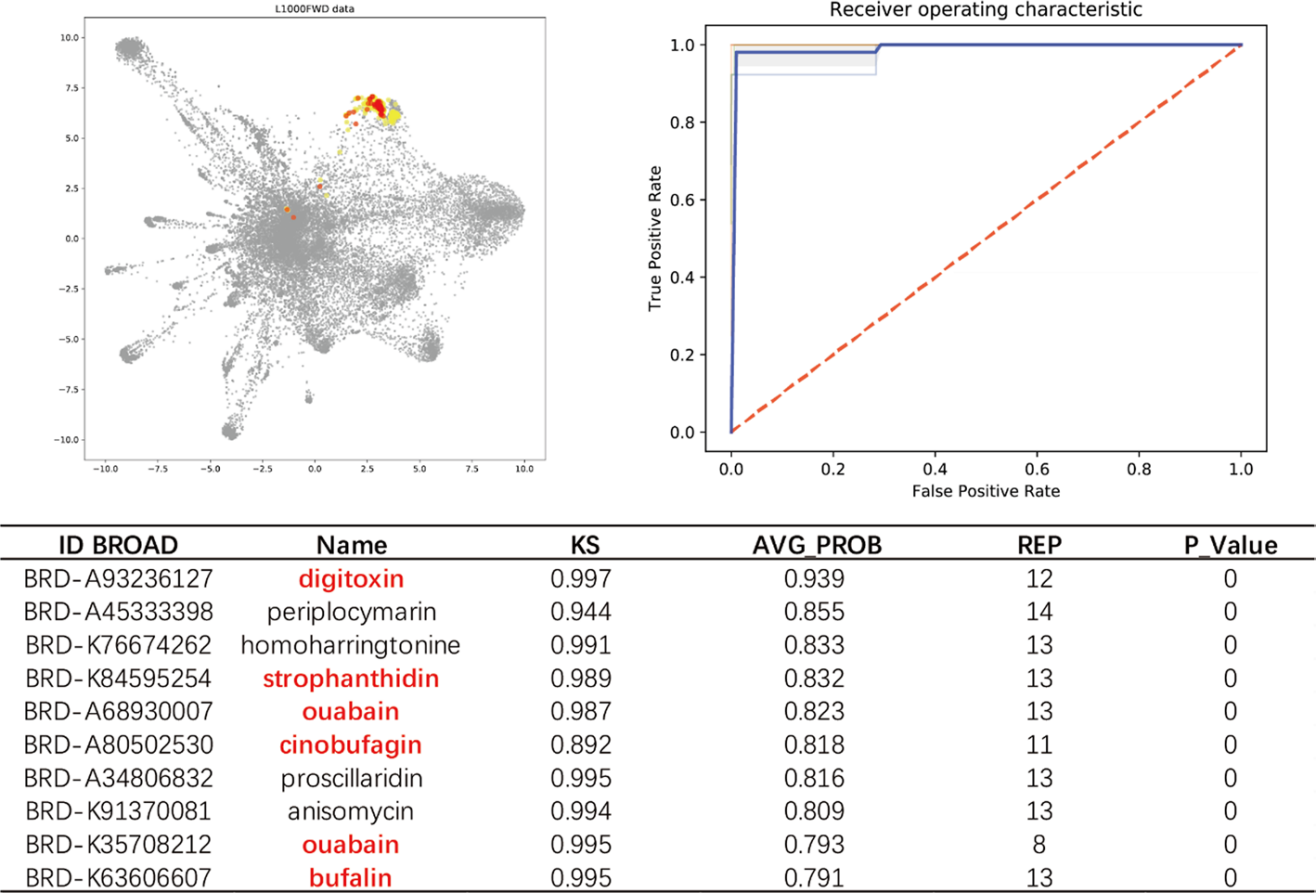
L1000FWD visualization data shows a trained Na+/K+-ATPase inhibitor Digoxin (red points), its mean AUROC = 0.99, and correspondingly predicted top10 most similar drugs (yellow points). 5 of the top10 predicted drugs (red) are known Na+/K+-ATPase inhibitor

“Na+/K+_ATPase inhibitors” is one of the easiest prediction models because cardiac glycosides are cytotoxic molecules which can induced significant phenotypic variations, and therefore usually have very similar and reproducible transcriptional profiles. Both GSEA and GPAR can achieve high performance. For example, when using digoxin as the only “positive” drug, the *AUROC* of GSEA was 1, similarly, as shown in Fig. 2, GPAR reached *AUROC* = 0.99, and the top10 predicted drugs included 5 known Na+/K+_ATPase inhibitors.

**Fig. 3:**
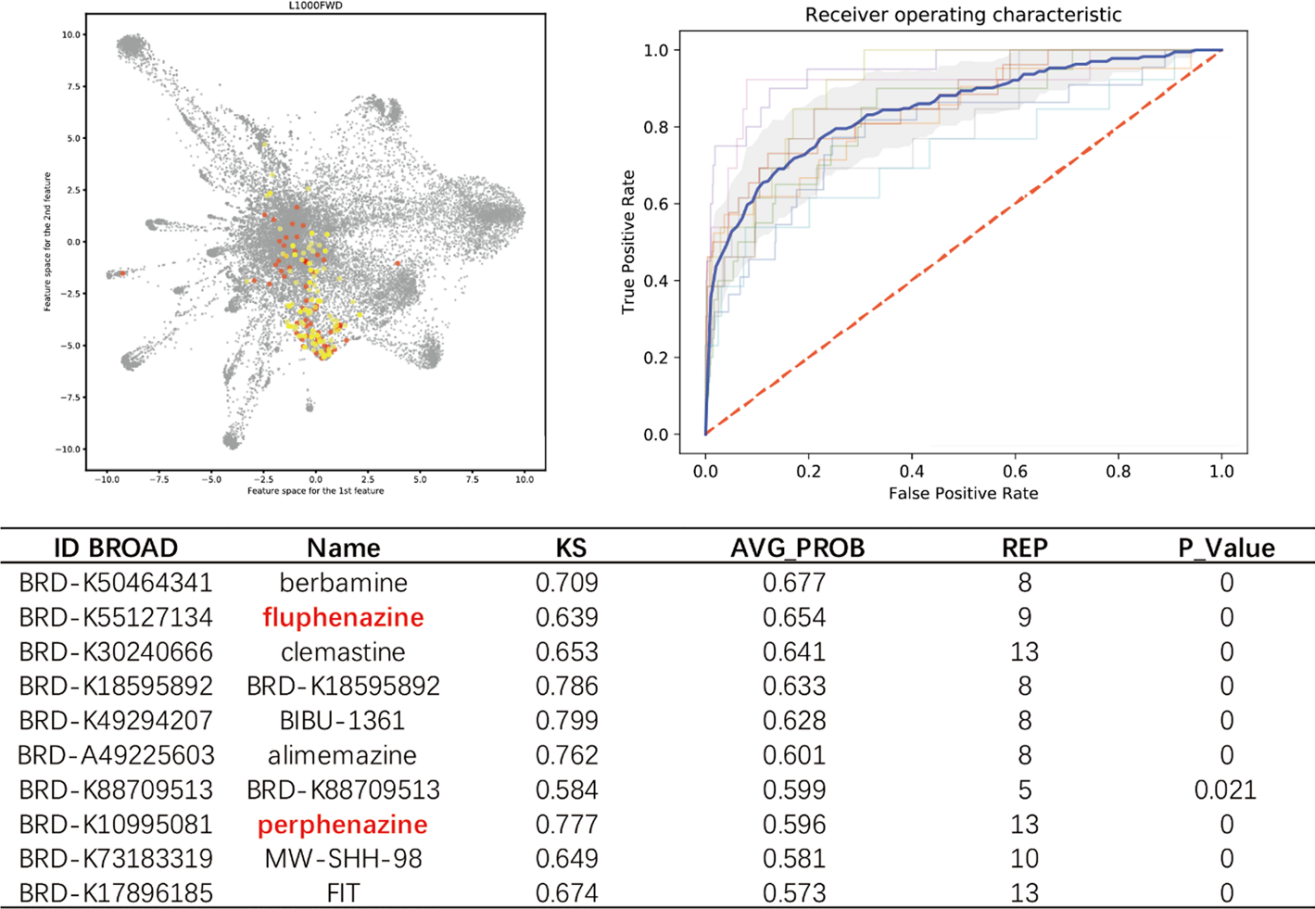
The output of serotonin transporter inhibitors includes the list of high score predicted compounds that may share the same MOA, visualization of trained 11 known serotonin transporter inhibitors (red) and top predicted potential drugs (yellow) and the *AUROC* of cross validation, its mean AUROC = 0.85. two of the top10 predicted drugs (red) are reported serotonin transporter inhibitors

For most MOAs with mild drug properties, GPAR also outperform GSEA. For example, serotonin transporter inhibitors are a class of antidepressants that mainly are targeted on central neural systems. When acting on cancer cell lines, serotonin transporter inhibitors usually have weak transcript signals and cell type variated signatures. We collect 11 known drugs: escitalopram, paroxetine, fluoxetine, clomipramine, sertraline, imipramine, milnacipran, doxepin, duloxetine, fluvoxamine and venlafaxine for training and predicting. Within the results, other known antidepressant drugs such as tetrindole, nortriptyline, lofepramine, indatraline that were not contained in training set were ranked in the top30. As shown in Fig. 3, the potential serotonin antagonist activities of Top10 predicted compounds, such as fluphenazine [30], perphenazine [31], were also reported. The *AUROC* of “serotonin transporter inhibitors” GPAR model was 0.85, whereas *AUROC* of GSEA was 0.75.

**Fig. 4:**
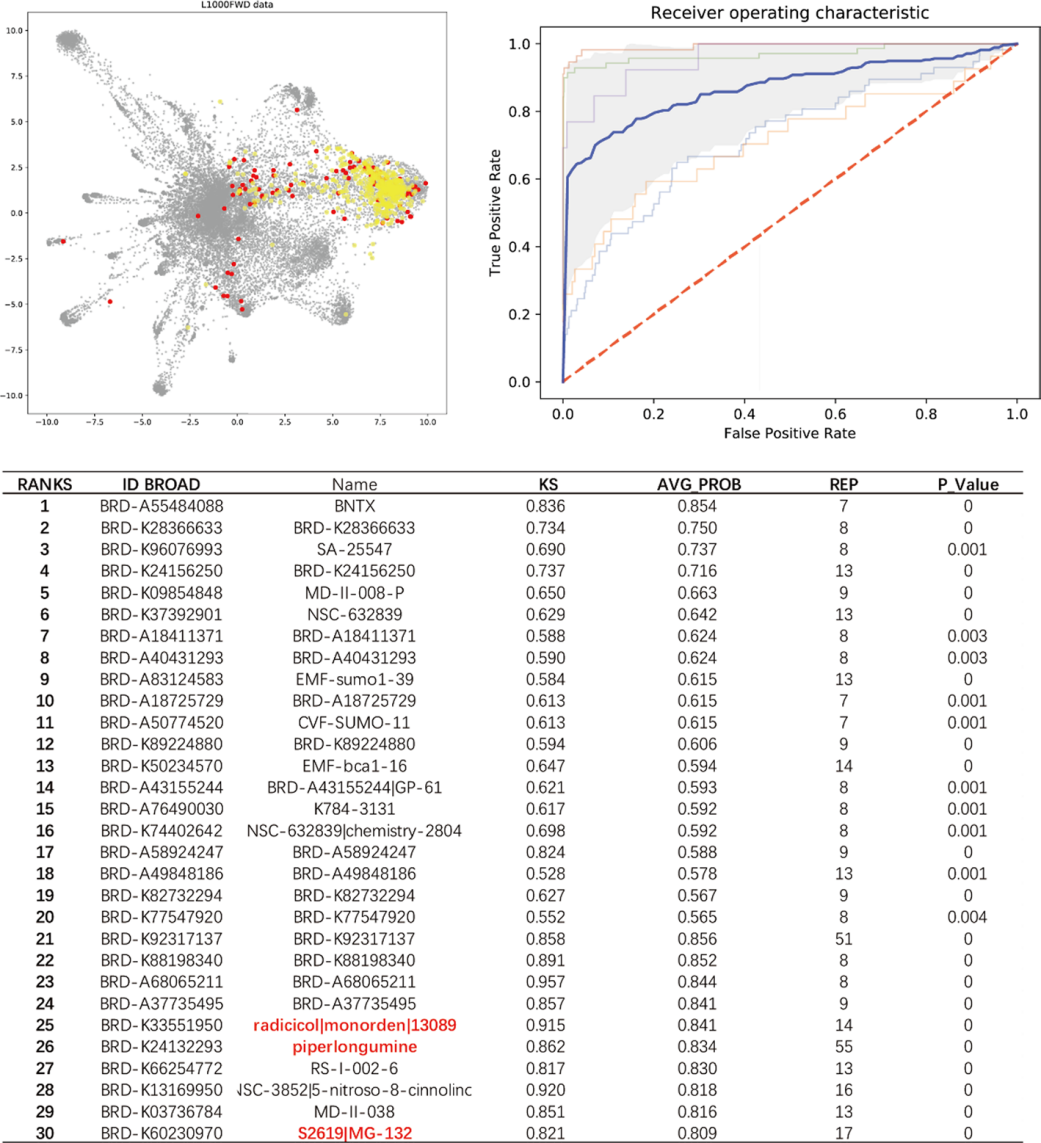
L1000FWD visualization data shows multiple trained NF-kB inhibitors (red points), and correspondingly predicted top30 potential positive compounds (yellow points), its mean AUROC = 0.87. Three molecules in table (red) are reported NF-kB inhibitors

Some MOAs, such as “NF-kB inhibitors”, were relatively difficult to be trained and predicted. As shown in Fig. 4, the signature points of NF-kB inhibitors were scattered in the visualization map, which means the signatures of NF-kB inhibitors generated from different cell types/dosages/time points are highly variant so that GSEA method only achieved *AUROC* = 0.66, while GPAR still achieved *AUROC* = 0.84. For the output results, there were 3 reported potential NF-kB inhibitors, e.g., Piperlongumine [32], radicicol [33], and MG-132 [34] in the top ranks.

In addition to drug set of general MOA types, high-throughput screening results (or user interested multiple drugs) are available. In recent days, an ongoing epidemic of coronavirus disease 2019 (COVID-19), caused by the new coronavirus, has caused a big problem worldwide. Although the World Health Organization has stated that preliminary identification of a novel virus in a short period of time is a notable achievement and demonstrates China’s increased capacity to manage new outbreaks, unfortunately the COVID-19 patients are suffering from the shortage of effective therapeutic drugs. Researchers are already trying antivirals widely used to treat HIV or other coronavirus, in hopes that they might be able to fight the COVID-19 as well. Many research institutes have devoted every effort to repurposing the potential old drugs for anti-virus through computation, while most of them are based on the docking approach, searching molecules that can minimize the binding energy with the new coronavirus targets such as spike protein or main protease. We showed that the GPAR here is also an efficient approach for searching similar drugs. Take Chloroquine as an example, it is a widely used lysosomotropic anti-malarial and autoimmune disease drug, which is one of the reported drugs that may inhibit COVID-19 in vitro [35], despite it has been shown to result in no clinical benefit in hospitalised patients by the large scale recovery trial as well as being no better than placebo at preventing symptomatic infection in another recent large-scale study [36]. Chloroquine was used as training set to train the prediction model and make further prediction. Two Nonsteroidal Antiinflammatory Drugs (NSAIDs), Oxaprozin and Niflumic acid, appeared among the Top10, sharing the same indication with Chloroquine for autoimmune diseases. More reliable calculated examples and comparisons could be found on the GPAR website and supporting Additional file 1: Tables S1–S2 in the supporting file.

## Discussions

In summary, we developed the GPAR method and online tool to connect MOAs with gene expression signatures, providing a simple and effective deep learning-based modeling and prediction method for drug researchers. From our results of *AUROC* and case study, GPAR showed high accuracy in most activity prediction. This online tool makes it easy for biologists and pharmarcologists to apply deep learning in modeling drug MOAs with expression profiles. The application scope is not limited to the pre-defined 103 kinds of drug MOAs, and users can even train their own model re-defined for activity screening. However, it is also found that the performance of GPAR in some activity prediction is not so good for some MOA predictions, and it may be due to the relativly small samples, the limitations of cell types (lack of relevant drug targets) or the drug property cannot be reflected at the transcriptome level [1, 20, 29]. The understanding of these problems is not intuitive due to the black box feature of deep learning, which should need more in-depth study.

Generally, full transcriptome data may be more conducive for machine learning. But L1000 expression data only directly measured 978 representative “Landmark genes”, which were selected from centroids of commonly co-regulated transcripts clusters in large scale microarrays analysis [20]; the rest ~ 10,000 genes expressions were obtained by computational inference [23], and hence inputting more genes may not be more informative. Besides, considering the cost in computational resource and “Curse of dimensionality” problems [37], it would be more easily for modeling the data with the smaller feature space. Therefore, the expression profiles of “Landmark genes” are more suitable for machine learning. Interestingly, we also noted that the models can achieve relatively high performance when leveraging only 100 Landmark genes (Additional file 1: Fig. S1), we then applied the feature selection algorithm of scikit-learn package [27] along with logistic regression to select 100 most important genes for each of 103 models from Landmark genes, and we noted that the selected genes of some MOAs were directly (HSP, PARP and HMGCR inhibitors) or functionally (MEK, MTOR inhibitors) related to their drugs targets (Additional file 1: Table S2), but there were still many MOAs that cannot related to the drug target. Nevertheless, although only leveraging 978 genes, we think the GPAR is still compatible with microarray-based gene expression dataset, For example, we have predicted the MOA of Traditional Chineses Medicine based on an independent dataset [38], containing more than 10,000+ gene expressions of 102 TCMs. In this research, Lv et al. validated that Nitidine chloride has the property of Topoisomerase Inhibition. When we queried Nitidine profiles (z-scores) in GPAR, the predictions is shown in Additional file 1: Table S3, which is consistent with their findings.

Here we note that the number of signatures among different cell lines are imbalanced [20], that is to say, there may be only few replicate experiments on some cell lines whereas dozens more on another. Besides, most signatures are generated from 9 out of 72 cell lines, and those imbalanced data might lead to bias on cell types. The aim of L1000 bioassay replicate is to measure the signature quality by consistency evaluation [9, 20], which provides a basis for us to select the high quality signatures and treat all 72 cell types more equally. So we reduced the sample size by selecting only one representative signature in each cell type for each molecule, and the reduced samples could ensure the quality of input data and meanwhile reduce the bias of sample numbers of different cell types.

More accurate taxonomy is benifical for identifying drugs with potential new targets or MOAs [39], here we use MOAs instead of a drug itself as training set label to avoid that the prediction model may not predict molecules with expected property, considering most drugs have more than one properties (many of them have not been identified), and prior knowledge based label can include multiple drugs [21], which is more conducive to extract the common characteristics of their signatures. The quality of training set labels is also important for supervised learning. Theoretically, any class label determined by prior knowledge about perturbagen, such as drug indications and side effects [16, 17], are available. But we think MOAs, especially the drug property at either targets or signaling pathways level, are more suitable as “positive set” labels. Because these drug properties such as therapeutics are too rough for drug classification, the mechanisms and expression profiles of drugs sharing same therapeutics may be quite different. For example, NHC proposed drugs for COVID-19 treatment have different mechanisms (anti-virus, anti-cytokine storm), and the *AUROC* for this model was only 0.63 ± 0.04. On the contrary, MOAs are more directly related to transcriptome data, and the drugs sharing the same MOAs might also show more similarities at transcriptome level. In order to further improve the quality of training set, we filter the “positive” drug set by repeating cross validation process to exclude the drugs whose signatures significantly decrease the mean *AUROC*. For example, six molecules were originally annotated “Heat Shock Proteins (HSP) inhibitor” including VER-155008, alvespimycin, geldanamycin, PU-H71, tanespimycin, BIIB021; but cross validation result showed that VER-155008 significantly decreased the *AUROC* from average 0.9 to 0.7. It is further found that VER-155008 is the only molecule among the training set that is targeted on HSP70 (a subtype of HSP receptor) whereas the others targeted on HSP90, showing that cross validation can get rid of some training sets that may be mislabeled.

## Conclusions

On the basis of GPAR method, more future additional signatures incorporating a broader taxonomic representation of drug perturbagens and cell-type diversity, together with in vivo data, can be incorporated into the GPAR models to improve MOA characterization and feature identification. A larger scale of transcriptome resource combing with heterogeneous dataset [39] will enable MOAs to be modeled with higher accuracy, sensitivity and reliability, and cover more subtypes of drug targets and cell types. GPAR could lead to unexpected connections and generate biological hypotheses for in-depth experimental validations, which could eventually facilitate the understanding of MOAs of new molecules or side effect of approved drugs. In conclusion, large scale perturbagen data serves good resources for machine learning, and GPAR provides a more powerful connection of expression signatures and MOAs, which could get the high accuracy in modeling MOAs and querying signatures, and facilitate drug repurposing opportunities.

## Supplementary Information

**Additional file 1:** Supplementary tables and figures.

## Data Availability

See 'Availability of materials and data' section for more information. These data can be obtained free of charge via http://gpar.idrug.net.cn/

## Abbreviations

MOA: Mechanism of action
ROC: The receiver operating characteristic curve
PRC: The precision-recall curve
AUROC: Area under the ROC
AP score: Average precision score of PRC
KNN: K-nearest neighbor
RF: RandomForest
NB: Naïve Bayes
GSEA: Gene set enrichment analysis
ES: Enrichment score
DEGs: Differentially expressed genes

## Supplementary Information

**Supplementary information** accompanies this paper at 10.1186/s12859-020-03915-6 .
