# Supplementary material for: Modeling drug mechanism of action with large scale gene-expression profiles using GPAR, an artificial intelligence platform
Source: BMC Bioinformatics. 2021 Jan 7;22:17. doi: 10.1186/s12859-020-03915-6 (PMC7788535; doi:10.1186/s12859-020-03915-6)
Supplement: Supplementary file 1 — Additional file 1: Supplementary tables and figures. [file 12859_2020_3915_MOESM1_ESM.docx]

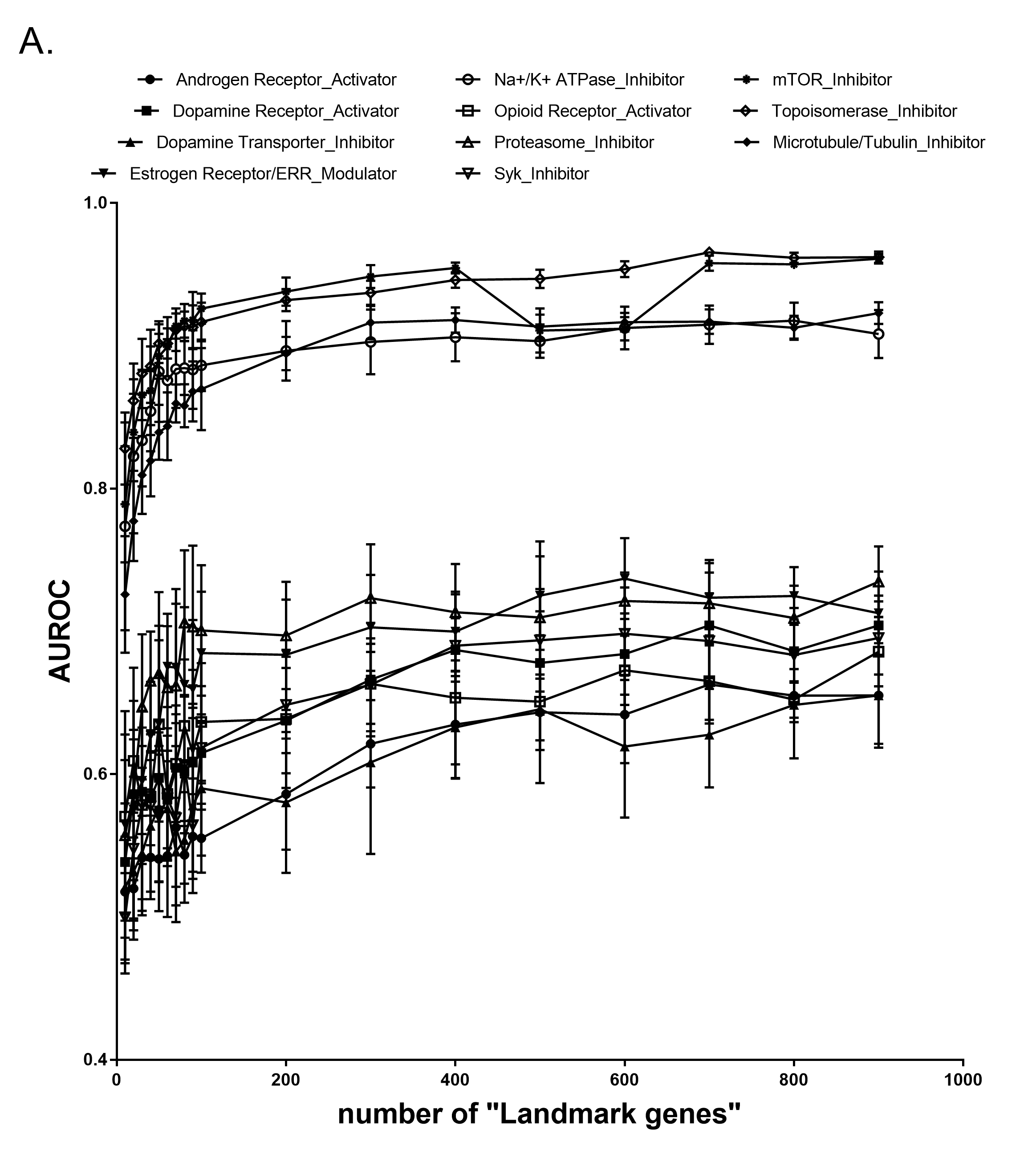


**Figure.S1.** The relations between AUROC and number of input features


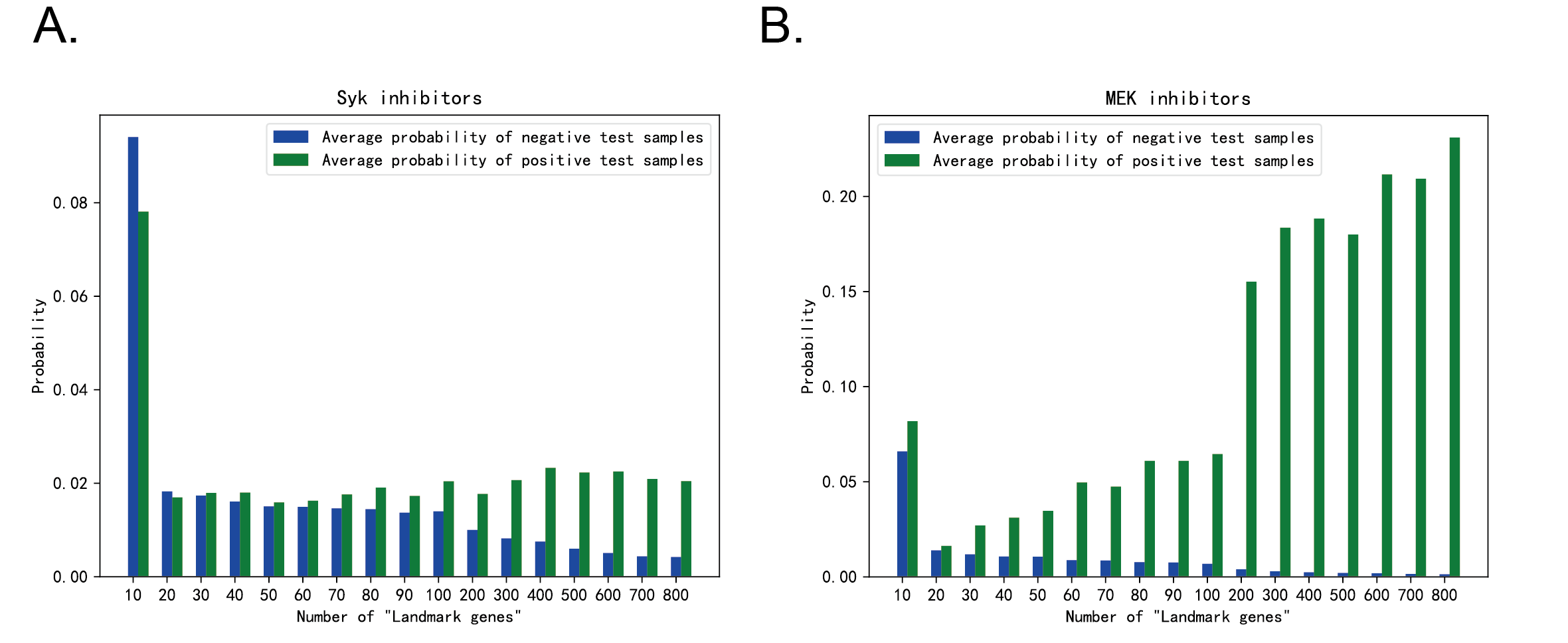


**Figure.S2.** With the increasing size of training features. the similarity between shared MOAs (A) Syk inhibitors and (B)MEK inhibitors has increased, as well as the distance between predefined positive and negative samples


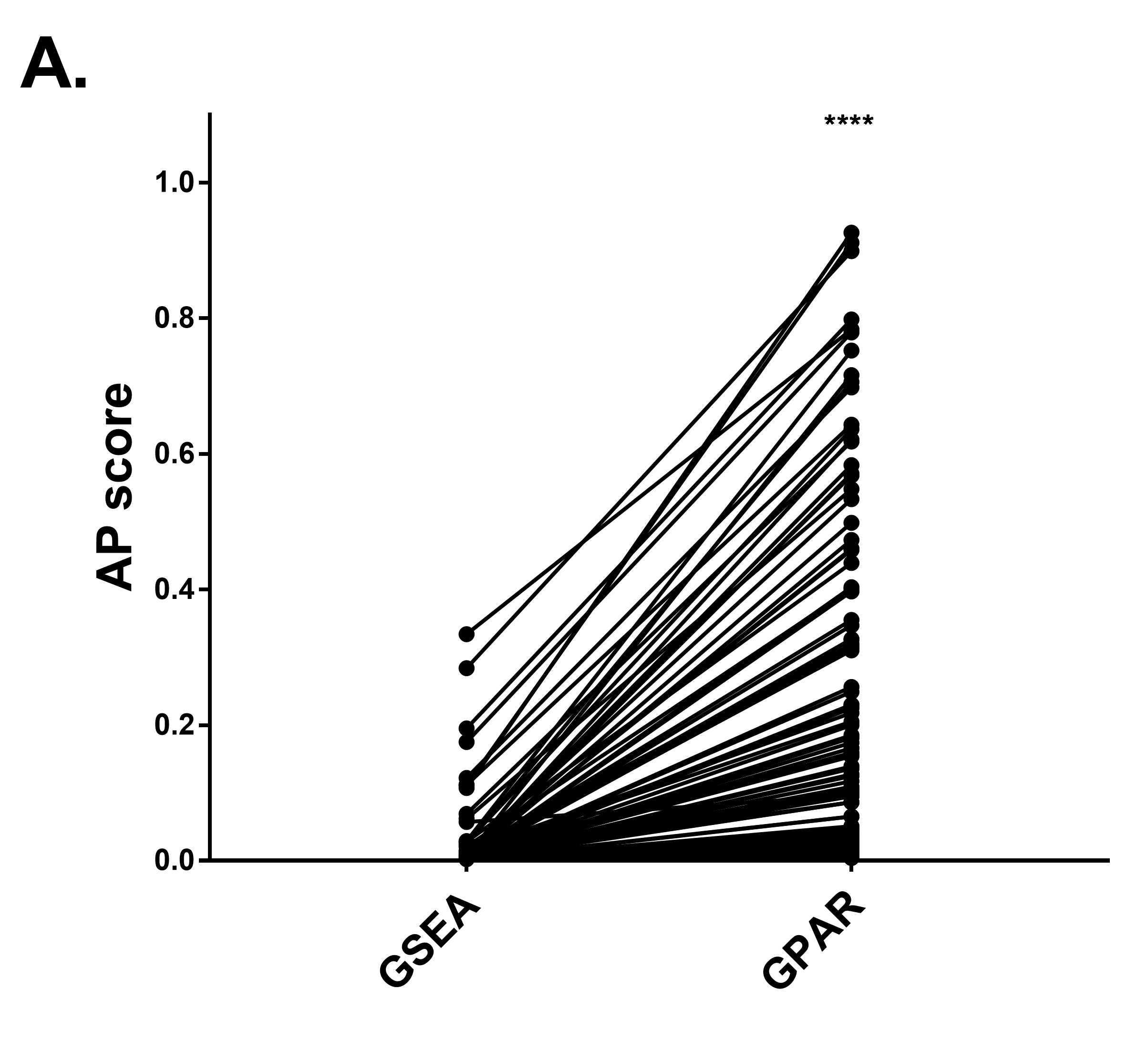


**Figure.S3.** AP score of PR curve comparison between GPAR and GSEA, ****：Wilcoxon matched-pairs signed rank test, vs GSEA, p<0.0001.


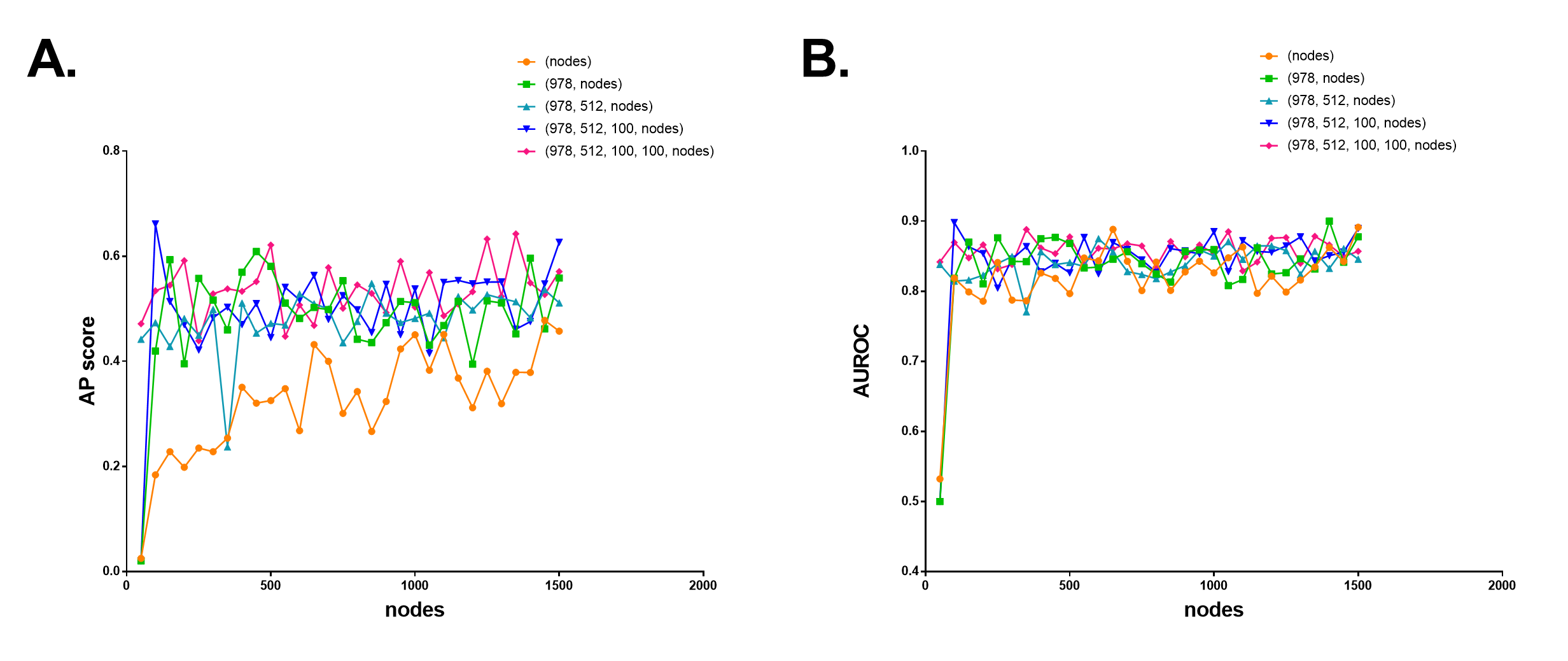


**Figure.S4.** both AUROC and AP score of ”MEK_Inhibitor” change with the hyperparameters “nodes” and “hidden layers”.


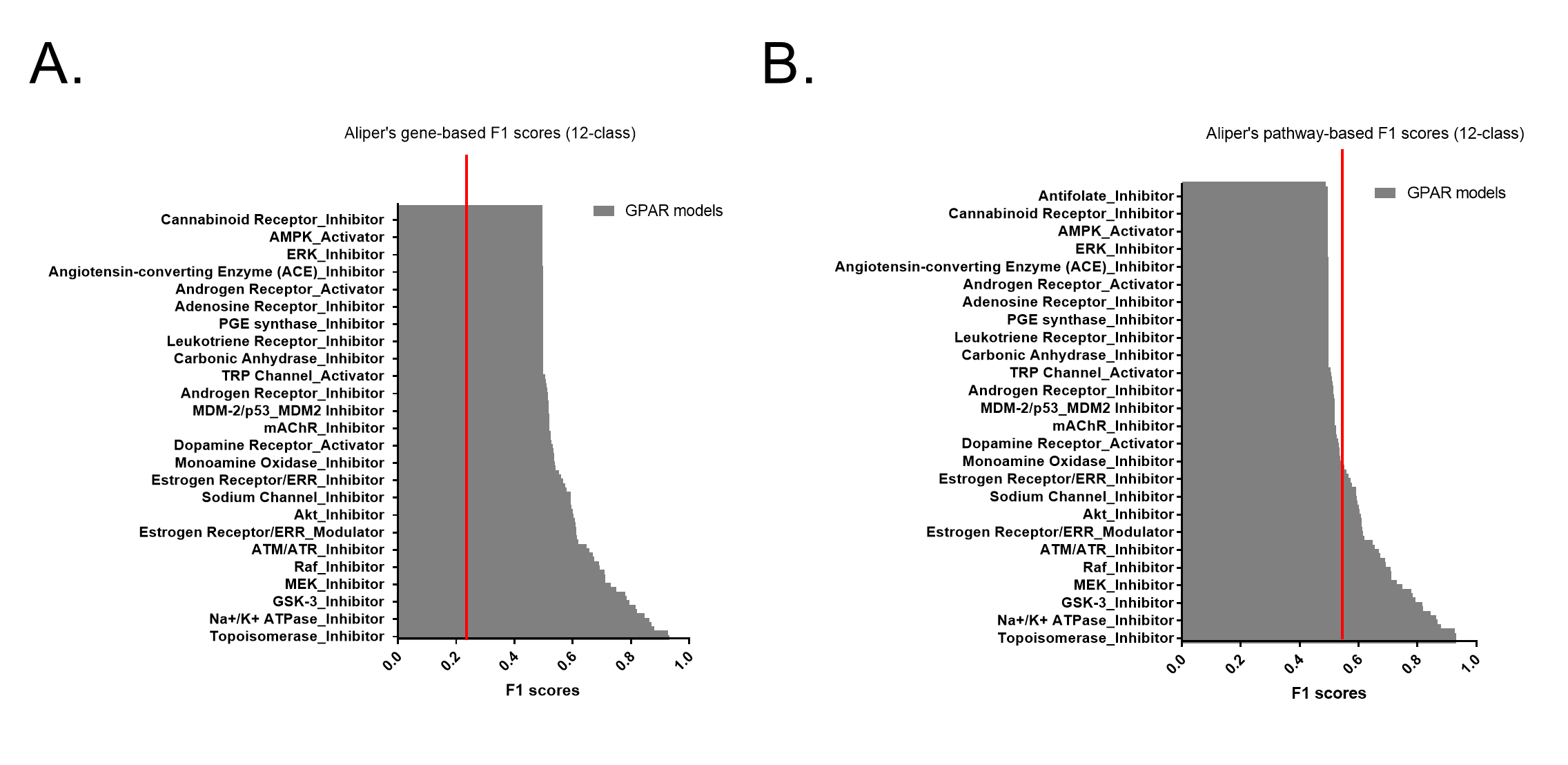


**Figure.S5.** F1 score of GPAR compared to that of Aliper’s (A) gene-based and (B) pathway-based models


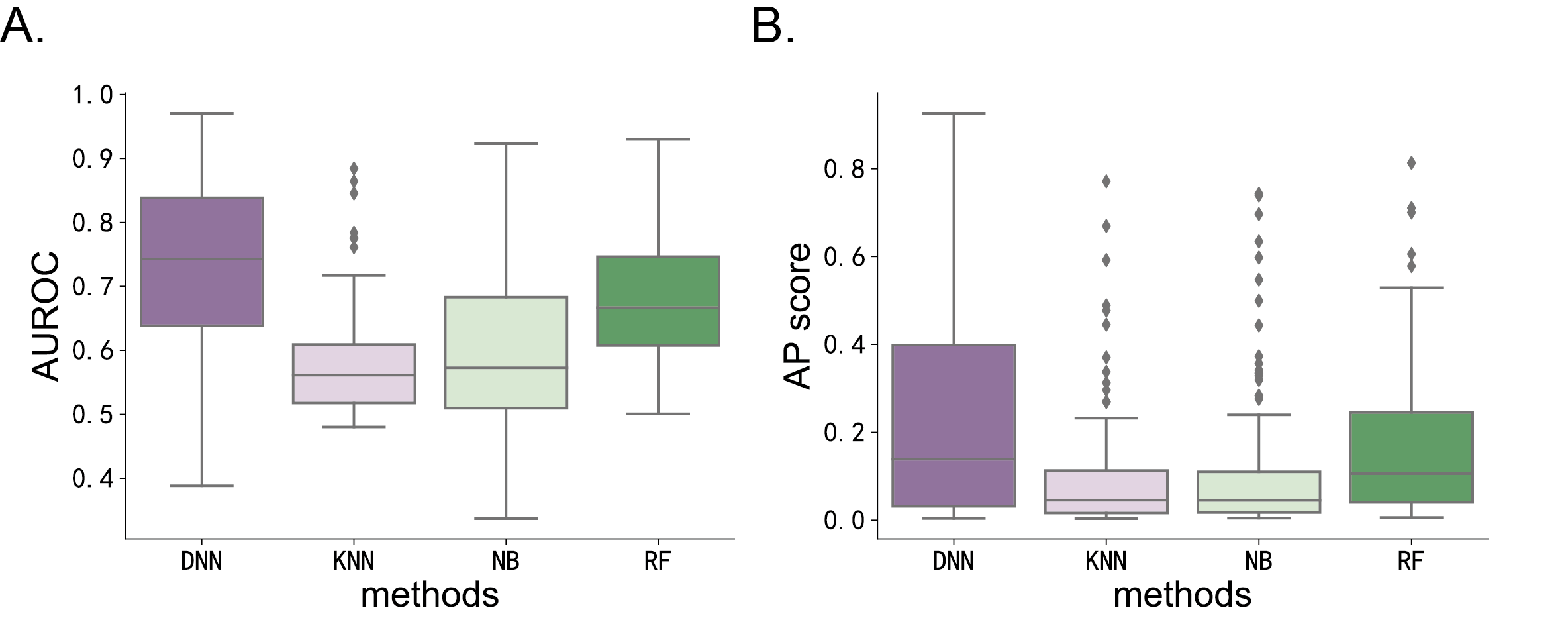


**Figure.S6. performance of four machine learning approach, including K-NearestNeighbor (KNN), RandomForest(RF) and Naïve Bayes (NB), on modeling 103 MOAs (**A) AUROC of ROC curve comparisons (B) AP score of PR curve comparison comparisons

**Table.S1.** The AUROC for GPAR and GSEA in modeling 103 MOAs.

| **MOAs** | **GSEA** | **GPAR** |
| --- | --- | --- |
| Topoisomerase_Inhibitor | 0.824 | 0.971 |
| Microtubule/Tubulin_Inhibitor | 0.857 | 0.948 |
| mTOR_Inhibitor | 0.897 | 0.940 |
| Na+/K+ ATPase_Inhibitor | 0.684 | 0.927 |
| MEK_Inhibitor | 0.764 | 0.920 |
| HSP_Inhibitor | 0.833 | 0.916 |
| Src_Inhibitor | 0.716 | 0.907 |
| PI3K_Inhibitor | 0.630 | 0.904 |
| GSK-3_Inhibitor | 0.920 | 0.900 |
| Glucocorticoid Receptor_Activator | 0.754 | 0.893 |
| HMG-CoA Reductase (HMGCR)_Inhibitor | 0.754 | 0.892 |
| PARP_Inhibitor | 0.815 | 0.886 |
| Raf_Inhibitor | 0.559 | 0.879 |
| Insulin Receptor_Inhibitor | 0.729 | 0.872 |
| EGFR_Inhibitor | 0.704 | 0.871 |
| 5-HT Receptor_Inhibitor | 0.540 | 0.870 |
| Adenylate Cyclase_Activator | 0.935 | 0.868 |
| ATM/ATR_Inhibitor | 0.792 | 0.864 |
| HDAC_Inhibitor | 0.688 | 0.862 |
| Dopamine Receptor_Inhibitor | 0.565 | 0.855 |
| STAT_Inhibitor | 0.702 | 0.855 |
| JAK_Inhibitor | 0.489 | 0.850 |
| Calcium Channel_Inhibitor | 0.566 | 0.846 |
| NF-kappaB_Inhibitor | 0.663 | 0.843 |
| CDK_Inhibitor | 0.725 | 0.841 |
| RAR/RXR_Inhibitor | 0.491 | 0.838 |
| Phosphodiesterase (PDE)_Inhibitor | 0.547 | 0.838 |
| Serotonin Transporter_Inhibitor | 0.750 | 0.833 |
| Histamine Receptor_Inhibitor | 0.514 | 0.821 |
| Sodium Channel_Inhibitor | 0.542 | 0.820 |
| Adrenergic Receptor_Inhibitor | 0.518 | 0.819 |
| Farnesyl Transferase_Inhibitor | 0.703 | 0.814 |
| Potassium Channel_Inhibitor | 0.541 | 0.813 |
| PPAR_Activator | 0.622 | 0.804 |
| Akt_Inhibitor | 0.570 | 0.795 |
| COX_Inhibitor | 0.574 | 0.793 |
| Bcl-2 Family_Inhibitor | 0.504 | 0.791 |
| Angiotensin Receptor_Inhibitor | 0.595 | 0.790 |
| Adrenergic Receptor_Activator | 0.537 | 0.790 |
| RAR/RXR_Activator | 0.825 | 0.783 |
| Estrogen Receptor/ERR_Activator | 0.545 | 0.779 |
| Estrogen Receptor/ERR_Inhibitor | 0.528 | 0.770 |
| VD/VDR_Activator | 0.775 | 0.767 |
| mAChR_Inhibitor | 0.571 | 0.765 |
| DNA Alkylator/Crosslinker | 0.654 | 0.762 |
| Syk_Inhibitor | 0.682 | 0.756 |
| TGF-beta Receptor_Inhibitor | 0.588 | 0.750 |
| Dopamine Receptor_Activator | 0.532 | 0.749 |
| ROCK_Inhibitor | 0.786 | 0.748 |
| Androgen Receptor_Inhibitor | 0.554 | 0.748 |
| Antifolate_Inhibitor | 0.655 | 0.745 |
| FGFR_Inhibitor | 0.554 | 0.743 |
| iGluR_Inhibitor | 0.510 | 0.739 |
| Glucocorticoid Receptor_Inhibitor | 0.696 | 0.731 |
| c-Met/HGFR_Inhibitor | 0.589 | 0.724 |
| Proteasome_Inhibitor | 0.596 | 0.721 |
| Estrogen Receptor/ERR_Modulator | 0.963 | 0.716 |
| Dopamine Transporter_Inhibitor | 0.899 | 0.710 |
| Androgen Receptor_Activator | 0.585 | 0.709 |
| Opioid Receptor_Activator | 0.484 | 0.707 |
| MDM-2/p53_MDM2 Inhibitor | 0.588 | 0.702 |
| Potassium Channel_Activator | 0.470 | 0.700 |
| 5-HT Receptor_Activator | 0.547 | 0.697 |
| Angiotensin-converting Enzyme (ACE)_Inhibitor | 0.547 | 0.696 |
| Prostaglandin Receptor_Activator | 0.525 | 0.694 |
| FAAH_Inhibitor | 0.471 | 0.692 |
| IKK_Inhibitor | 0.564 | 0.687 |
| GABA Receptor_Inhibitor | 0.617 | 0.687 |
| TNF Receptor_Inhibitor | 0.609 | 0.684 |
| Cytochrome P450_Inhibitor | 0.520 | 0.683 |
| MMP_Inhibitor | 0.573 | 0.680 |
| Adenosine Receptor_Inhibitor | 0.715 | 0.680 |
| nAChR_Activator | 0.381 | 0.668 |
| Progesterone Receptor_Inhibitor | 0.651 | 0.647 |
| Sigma Receptor_Activator | 0.588 | 0.644 |
| Tyrosinase_Inhibitor | 0.455 | 0.644 |
| FXR_Activator | 0.495 | 0.640 |
| Polo-like Kinase (PLK)_Inhibitor | 0.432 | 0.636 |
| ERK_Inhibitor | 0.608 | 0.630 |
| AChE_Inhibitor | 0.556 | 0.622 |
| CaMK_Inhibitor | 0.671 | 0.611 |
| Endothelin Receptor_Inhibitor | 0.435 | 0.601 |
| CCR_Inhibitor | 0.399 | 0.601 |
| DNA Methyltransferase_Inhibitor | 0.568 | 0.600 |
| Aldose Reductase_Inhibitor | 0.678 | 0.597 |
| Factor Xa_Inhibitor | 0.514 | 0.596 |
| TRP Channel_Activator | 0.507 | 0.588 |
| Leukotriene Receptor_Inhibitor | 0.712 | 0.587 |
| JNK_Inhibitor | 0.374 | 0.581 |
| P-glycoprotein_Inhibitor | 0.445 | 0.580 |
| AMPK_Activator | 0.456 | 0.578 |
| Carbonic Anhydrase_Inhibitor | 0.445 | 0.563 |
| Monoamine Oxidase_Inhibitor | 0.552 | 0.555 |
| Histone Methyltransferase_Inhibitor | 0.119 | 0.547 |
| PGE synthase_Inhibitor | 0.724 | 0.542 |
| Proton Pump_Inhibitor | 0.453 | 0.539 |
| Adenosine Receptor_Activator | 0.638 | 0.536 |
| Monoamine Transporter_Inhibitor | 0.911 | 0.501 |
| mGluR_Inhibitor | 0.400 | 0.486 |
| GABA Receptor_Activator | 0.671 | 0.481 |
| Opioid Receptor_Inhibitor | 0.633 | 0.477 |
| Cannabinoid Receptor_Inhibitor | 0.322 | 0.449 |
| Phosphatase_Inhibitor | 0.399 | 0.388 |

**Table.S2.** feature selection of serveral MOAs, in which some genes (red) were directly or functionally related to drugs targets

| **MOAs** | **five most important features** |
| --- | --- |
| MEK inhibitors | DUSP4, DUSP6, CCND1, ITGB5, IER3 |
| MTOR inhibitors | DDIT4, MYC, KIF14, BNIP3L, EED |
| HSP inhibitors | TMEM97, SPR, HSPD1, ATP11B, MTF2, |
| PARP inhibitors | PLCB3, TIPARP, C2CD2, RRP8, GATA2 |
| HMGCR inhibitors | TSC22D3, RHOA, HMGCR, CSNK1E, TCTA |

**Table.S3.** Nitidine MoA predictions

| **predicted MoAs** | **Probabilty** |
| --- | --- |
| Topoisomerase_Inhibitor | 1.00 |
| DNA Alkylator/Crosslinker | 1.00 |
| MDM-2/p53_MDM2 Inhibitor | 1.00 |
| Calcium Channel_Inhibitor | 1.00 |
| CDK_Inhibitor | 0.91 |
| GSK-3_Inhibitor | 0.84 |
| IKK_Inhibitor | 0.81 |
| Dopamine Receptor_Inhibitor | 0.52 |
| Syk_Inhibitor | 0.33 |
| PARP_Inhibitor | 0.25 |
